# Riboflavin attenuates tartrazine toxicity in the cerebellar cortex of adult albino rat

**DOI:** 10.1038/s41598-022-23894-3

**Published:** 2022-11-11

**Authors:** Omnia I. Ismail, Noha A. Rashed

**Affiliations:** Lecturer of Human Anatomy and Embryology, Human Anatomy and Embryology Department, Faculty of Medicine, Assiut University, Assiut, 71515 Egypt

**Keywords:** Cell biology, Neuroscience, Anatomy

## Abstract

Tartrazine is a synthetic yellowish dye considered one of the most common food colorants. Extensive usage of tartrazine in humans led to harmful health impacts. To investigate the impact of tartrazine administration on the cerebellum and to assess the potential role of riboflavin co-administration in the adult male albino rat. Four groups of adult albino rats were included in this study. Group I was supplied with distilled water. Group II was supplied tartrazine orally at a dose of 7.5 mg/kg BW dissolved in distilled water. Group III was supplied with tartrazine at the same previously mentioned dose and riboflavin orally at a dose of 25 mg/kg BW dissolved in distilled water. Group IV was supplied with riboflavin at the same previously mentioned dose. The study was conducted for 30 days then rats were sacrificed, weighted and the cerebella extracted and handled for light, ultrastructural and immunohistochemical evaluation. It was found with tartrazine treatment focal areas of Purkinje cell loss leaving empty spaces, a broad spread of neuronal affection to the degree of the disappearance of some of the granular cells, reduced the thickness of the molecular and granular layers, and strong positive GFAP immunoreactions. With riboflavin coadministration restored continuous Purkinje layer with normal appeared Purkinje cells, but some cells were still shrunken and vacuolated as well as the molecular and granular cell layers appeared normal. Tartrazine had deleterious effects on the cerebellar cytoarchitecture, and riboflavin co-administration alleviated these neurotoxic effects.

## Introduction

Tartrazine (E 102, FD, and C Yellow) is a synthetic yellowish dye considered one of the most common food colorants which are extensively utilized in the food, leather, cosmetic, and textiles industries as well as in the drug capsules like antiacids and vitamins especially in many developing countries^1^. Tartrazine is absorbed by the intestinal epithelium and metabolized via the microflora and perhaps through hepatic or intestinal wall mammalian azo reductase to sulphanilic acid which has a potentially carcinogenic capability^2^. Oral administration of tartrazine leads to the excretion of equal amounts of sulphanilic acid which is partially conjugated in the rat, rabbit and man.

Many species, including rats and humans, undergo this biotransformation pathway, which causes a variety of illnesses^3^.The findings on the toxicokinetic of tartrazine in humans seem to be in line with those found in laboratory animals^4^.

The acceptable daily intake of tartrazine for human consumption reported by the World Health Organization and Joint FAO/WHO expert committee on food additives **(**JECFA) is about 7.5 mg/kg based on a 2-year feeding study in rats that determined a no-observed-adverse-effect level (NOAEL) of 750 mg/kg body weight/day^5,6^**.**

Also, teir 3 refined exposure study estimates that the 95th or 97.5th percentile exposure levels for tartrazine might reach up to 7.3 mg/kg body weight/day when considering solely dietary consumption (which is not the only source of exposure)^7^.

The average daily consumption of tartrazine in the general population ranges from 0.000671 to 14 mg/person, depending on the nations and study types. This variation may also be explained by regional variations in dietary practices, as, for instance, dye consumption is likely lower in Japan than it is in America^4^.

Moreover, the interaction between several drugs further complicates the description of the necessary safety limits for human intake of tartrazine^3^.

The former literature reported that tartrazine causes various behavioral changes such as hyperactivity, irritability, restlessness, and sleep disorder in children. Also, extensive usage of tartrazine in humans led to harmful health impacts such as thyroid cancer, asthma, eczema, migraines, genotoxicity, liver and kidney impairment, and infertility^2,5,8,9^.

The toxic effects of tartrazine exposure to humans are of serious concern because of its extensive usage in the sweets and beverages to provide color to the products to make them more attractive. However, there was insufficient research has been carried out to determine tartrazine effect on the architecture of the cerebellum.

The cerebellum has a vital role in the control of posture and coordination of movement. Also, it involves various nonmotor intellectual functions including attention, learning, sensory differentiation, and memory. Thus, a destructive lesion of the cerebellum disrupts motor coordination and impairs balance^10^. The astrocytes are specialized glial cells present along the entire central nervous system and act as a macrophage**.** The activation of astrocytes is an indicator of neurodegeneration and exposure to any neurotoxic substance^11^; hence it is significant to study them to evaluate tartrazine toxicity.

Riboflavin (vitamin B2) is a water-soluble vitamin and one of the vitamin B complexes that naturally exists in a diversity of plant and animal sources such as meat, liver, eggs, dairy products, and dark green leafy vegetables^12^. Riboflavin has been considered a promising antioxidant, anti-inflammatory, antineoplastic, and immune-modulatory agent ^13^. In addition, it is essential for several cellular functions and enzymatic processes. In the nervous system, riboflavin is vital in myelin synthesis^14^. Moreover, it is utilized for the management of many nervous clinical diseases such as depression and migraine^15^.

Based on the previous data, we aimed to investigate the impact of administration of tartrazine on the cerebellar cortex and to assess the potential role of riboflavin co-administration in the adult albino rat.

## Material and methods

### Experimental animals

The study was conducted on forty adult male albino rats (three months aged weighing 200–220 g) which were purchased from the Animal House at the Faculty of Medicine, Assiut University, Assiut, Egypt. The rats hosted under 12 h light/dark cycles and relative humidity in clean, well-ventilated cages with free access to food and water. Animal manipulations followed the globally recognized criteria for the Care and Use of Laboratory Animals and were authorized by the ethics committee at the Faculty of Medicine, Assiut University, Assiut, Egypt.

### Chemicals

El-Gomhouria Company for Trading Chemicals and Medical Appliances, Assuit, Egypt, provided Tartrazine E 102 AR and Riboflavin in powder form. All other chemicals and reagents were obtained from standard commercial suppliers.

### Experimental design

The experimental albino rats were partitioned into equal four groups. Each group consisted of 10 rats.

Group I (negative control group) was supplied with distilled water.

Group II (Tartrazine treated group) was supplied with tartrazine dissolved in distilled water at a dose of 7.5 mg/kg body weight as mentioned by^16^.

Group III (Tartrazine + Riboflavin treated group) was supplied with tartrazine at the same previously mentioned dose and Riboflavin dissolved in distilled water at a dose of 25 mg/kg as mentioned by^13^. Numerous studies on different doses of tartrazine and riboflavin were conducted. The current dose of tartrazine was chosen according to the acceptable daily intake of tartrazine for human consumption^5^.

The current dose of riboflavin was chosen according to the prior studies that demonstrated that oral riboflavin treatment at 25 mg/kg BW to rats activated superoxide dismutase which is one of the antioxidant enzymes that can hinder oxidative stress conditions^17,18^.

Group IV (positive control group) was supplied with Riboflavin at the same previously mentioned dose.

For 30 days, the rats were given a daily dose of the drugs by a gavage needle. A suitable-sized oral feeding needle inserted into the oesophagus of conscious restrained animal while its body and head were in a vertical, straight line to allow easier passage of the feeding needle. The ball tip of the needle inserted into the mouth and rose up the syringe and needle to softly pressing those against the palate then passed gently through oesophagus and finally the drugs administered. All experimental animals were well-being with on abnormal signs during the experiment, and no animals were excluded from it.

When the experiment is finished, the rats were weighed and anesthetized with intraperitoneal injection of 25 mg/kg sodium thiopental. Intra-cardiac perfusion was performed using butterfly needle inserted into the left ventricle of the heart. Initially the intravascular perfusion by saline solution (0.9% NaCl) to wash out the blood from the body then 2.5% glutaraldehyde in 0.1 mol/L cacodylate buffer (pH. 7.3) used for cerebellar partial fixation.

Each rat was decapitated via straight sharp scissors then the cap and lateral walls of the cranium were gently removed. The dura mater was incised and raised then the falx cerebri was pulled out and tentorium cerebelli was trimmed. The cerebella specimens were carefully extracted. The right hemisphere of each cerebellum of each animal was used for light microscopic and immunohistochemical study and the left hemisphere was used for electron microscopic examination.

### Light microscopic study

The cerebella specimens were preserved by embedding them in 10% formaldehyde, undergone a dehydration process with alcohol series then preparation of the paraffin blocks was done. The paraffin blocks were sectioned with 5 μm thin sections by a microtome (MICROM HM 340E, WALLDORF, Germany). The sections were stained with the gallocyanin-chrome alum stain^19^.

### Immunohistochemical study

Immunohistochemical investigation of glial fibrillary acidic protein (GFAP) expression was performed. The deparaffinization process of tissue sections was done then ethanol and phosphate-buffered saline (PBS) was applied to rehydrate. 10% hydrogen peroxidase in filtered water was added to the tissue sections to block endogenous peroxidase activity. Then, the restoration of the antigen was done via boiling the slides with10 mM of citrate buffer (pH 7) for ten minutes. The primary antibody anti- GFAP (dilution 1:150, Catalog#MA5-17139 Thermo Fisher Scientific, CA, USA) was applied to the slides for a night in humidity at room temperature. The next morning, the slides were washed then a secondary antibody (anti-goat, dilution 1:100, Abways) was added for 120 min. Finally, the sections were stained with a diaminobenzidine chromogen kit (Beyotime Biotechnology Co, LTD.) and then counterstained with Mayer's haematoxylin, dehydrated, cleared by xylene and covered. Non-immune serum was used in place of the primary antibodies in negative control sections^20^. Under a light microscope, the sections were examined at Human Anatomy and Embryology Department, Faculty of Medicine, Assiut University, Assiut- Egypt.

### Electron microscopic study

The specimens of cerebella were cut in the sagittal plane into minor pieces and then poured into the primary fixation that consisted of cold buffered 2.5% glutaraldehyde + 4% formaldehyde. The primary fixation was washed by adding distilled water to the specimens 3 times in 10 min. 1% Osmium Tetroxide was applied to the specimens for 1 h then flushed by adding distilled water 3 times. The specimens were dehydrated then embedded in epoxy resin then polymerized. The semithin sections (1 micron) were cut via the ultra-microtome then staining with the toluidine blue was done. Ultra-thin sections (0.1 micron) were trimmed via the Leica ultracut (Deerfield, IL, USA) then staining with uranyl-acetate and lead citrate was carried out^21^. The transmission electron microscope (TEM) ("Jeol" E.M.-100 CX11; Japan) was used to examine ultra-thin sections at the Electron Microscopic Unit of Assiut University, Assiut- Egypt.

### Morphometric assessment and statistical analysis

For morphometric evaluation, the computerized image analyzer system software (Leica Q 500 MCO; Leica, Wetzlar, Germany) joined to a camera connected to a Leica universal microscope at Human Anatomy and Embryology Department, Faculty of Medicine, Assiut University, Assiut, Egypt was used to evaluate the thickness of the molecular and granular layers using gallocyanin-stained sections. Additionally using the cell counter plugin in Image J software, we calculated the linear density of the Purkinje cells in Nissl-stained sections and the area percent of GFAP positive expression in GFAP immunostained sections at magnification ×400. Ten readings from ten non-overlapping fields from each specimen in each studied group were taken. The percent of damaged myelinated axons in all experimental group was calculated using electron microscopic examination.

For comparison between the studied groups, the morphometric data were compared by one-way analysis of variance (ANOVA) followed by Tukey's post hoc test via SPSS program version 16 (SPSS Inc., Chicago, USA). Mean value ± standard deviation was calculated and used to express the data. It was considered significant if the P-value (probability value) was < 0.05.

### Ethics approval and consent to participate

Ethical approval was obtained from the Ethical Committee of Faculty of Medicine, Assiut University, Egypt. All methods were performed in accordance with the relevant guidelines and regulations and in compliance with ARRIVE guidelines for the care and use of experimental animals by the committee for supervision of Experiment on animals (CPCSEA) and the National Institute of Health NIH Protocol.

## Results

Examination of the sections in the negative and positive control groups revealed the same normal histological structure. So, the mentioned results titled under the control group represented those of both control groups.

### Light microscopic results

**Figure 1:**
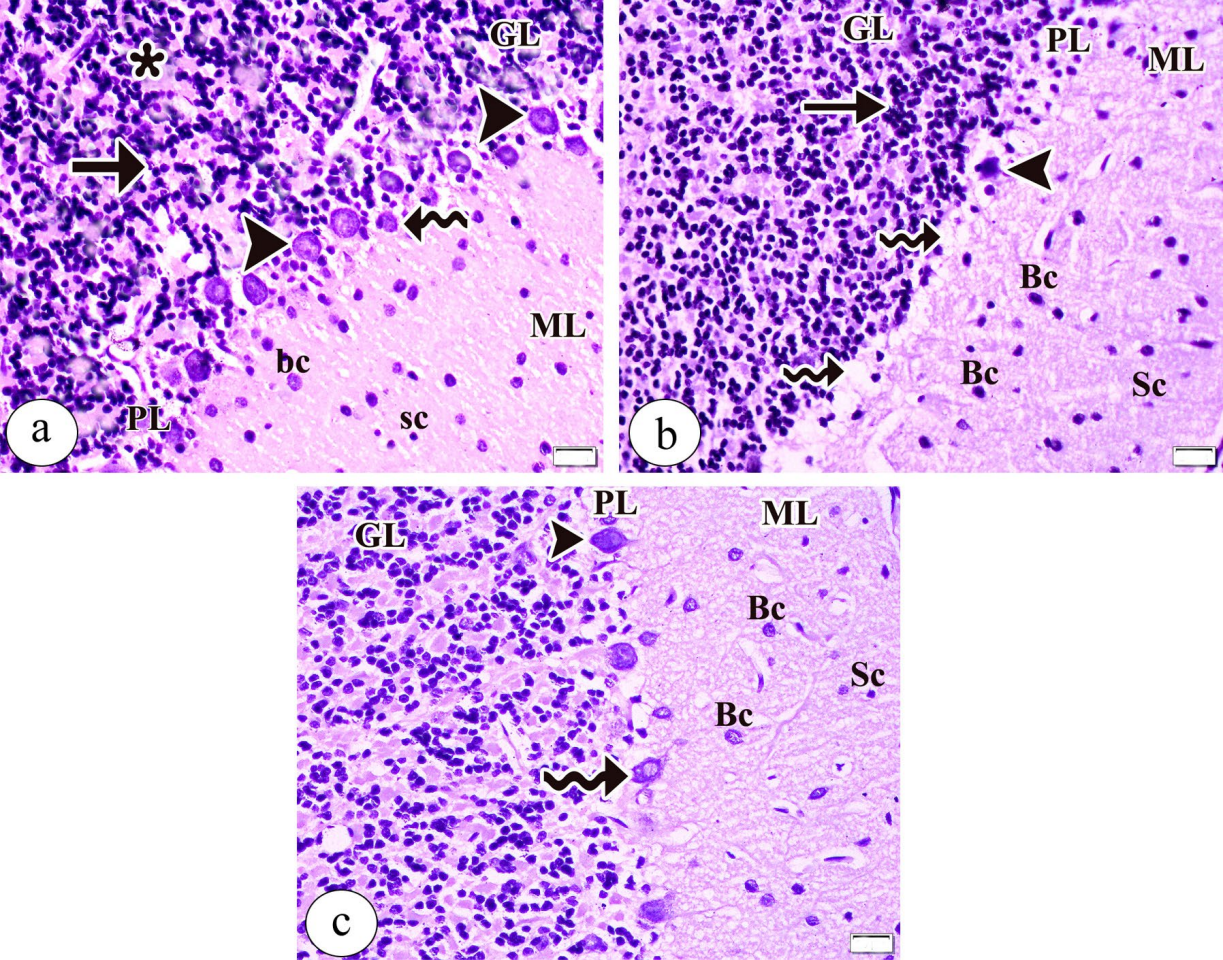
A photomicrograph of the sections of the cerebellar cortex in (**a**) the control group showing the distinct layers; the molecular (ML), Purkinje cell (PL), and granular cell (GL) layers. The molecular layer (ML) is consisting of small-sized stellate (sc) and large-sized basket (bc) cells. The Purkinje cell layer (PL) is formed of a single line of the large flask-shaped Purkinje cells (arrowhead) with open face vesicular nucleus and Nissel's granules as well as small Bergmann protoplasmic astrocytes (curved arrow). The granular cell layer (GL) has numerous small rounded packed dense cells (arrow) and the cerebellar islands (asterisk). (**b**) the tartrazine treated group showing the Purkinje cell layer (PL) have Purkinje cell loss in concentrated areas leaving empty spaces (curved arrow) and dense shrunken Purkinje cells with a pyknotic nucleus and (arrowhead). The granular cell layer (GL) shows clumped dense granular cells (arrow). (**c**) the tartrazine + riboflavin treated group showing regain of continuous Purkinje cell layer (PL) with normal appeared Purkinje cell (arrowhead) but some Purkinje cell (curved arrow) is still shrunken and vacuolated. The molecular (ML) and granular cell layer (GL) appear normal. (Gallocyanin stain., × 400, scale bar = 20 μm).

Examination of the Gallocyanin stained sections in the control group demonstrated histologically normal architecture of the cortex of cerebellum. The cortex was formed of three layers from outside to inside; molecular, Purkinje, and granular layers. The molecular layer consisted of small-sized stellate and large-sized basket cells. The layer of Purkinje cell formed of a single line of the large flask-shaped Purkinje cells with open face vesicular nucleus and Nissel’s granules at the intersection of the molecular and the granular layers as well as small Bergmann protoplasmic astrocytes. The granular cell layer had numerous small rounded packed dense cells and the cerebellar islands (Fig. 1a). On contrast, the tartrazine treated group showed the Purkinje cell layer with Purkinje cell loss in concentrated areas leaving empty spaces. In addition, dense shrunken Purkinje cells with pyknotic nuclei were detected. The granular cell layer revealed clumped dense granular cells (Fig. 1b). Surprisingly, examination of the sections in the tartrazine + riboflavin treated group demonstrated regain of continuous Purkinje cell layer with normal appeared Purkinje cell, but some Purkinje cells were still shrunken and vacuolated. The molecular and granular cell layer appeared more or less normal (Fig. 1c).

**Figure 2:**
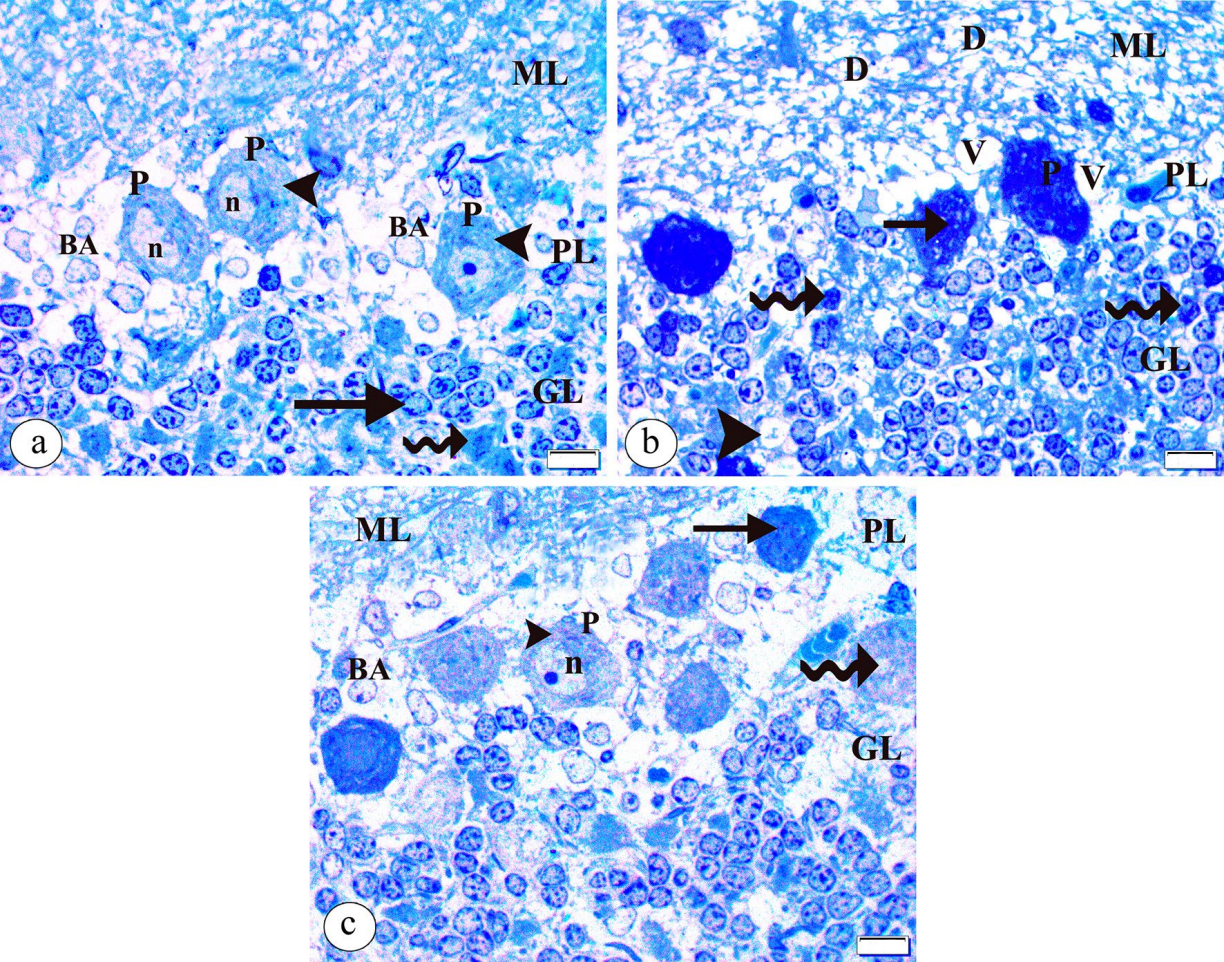
A photomicrograph of the semithin sections of the cerebellar cortex in (**a**) the control group showing the molecular layer (ML), Purkinje cell layer (PL), and granular cell layer (GL). The normal appeared Purkinje cell (P) with central vesicular infolded nucleus (n) and homogenous cytoplasm (arrowhead) encircled by Bergmann protoplasmic astrocytes (BA) with granular chromatin. The granule cells (arrow) have darkly stained nuclei with granular chromatin and the cerebellar islands (curved arrow) are noticed in between. (**b**) the tartrazine-treated group showing degenerated areas (D) in the molecular layer (ML). The Purkinje cell layer (PL) shows dark Purkinje cells (P) with an irregular outline and an invisible nucleus. Shrunken Purkinje cell (arrow) also appears. The granular cell layer (GL) shows deeply stained cells (curved arrow), vacuolated areas (V), and vacuolated cerebellar island (arrowhead). (**c**) the tartrazine + riboflavin treated group showing the molecular layer (ML) and granular cell layer (GL) appear normal. The Purkinje cell layer (PL) shows normal Purkinje cell (P) with vesicular nucleus (n), prominent nucleolus, and homogenous cytoplasm (arrowhead) surrounded by Bergmann protoplasmic astrocytes (BA). Some deeply stained Purkinje cells (arrow) and downward displaced Purkinje cells (curved arrow) are also seen. (Toluidine blue, ×1000, scale bar = 10 μm).

In toluidine blue-stained semithin sections of the control group, the Purkinje layer normally appeared and showed the Purkinje cells with a central vesicular infolded nucleus and homogenous cytoplasm surrounded by Bergmann protoplasmic astrocytes with granular chromatin. The granular cell layer showed the granule cells having dark nuclei and granular chromatin in addition to the cerebellar islands in between (Fig. 2a). Contrarily, the tartrazine-treated group displayed degenerated areas in the molecular layer. The Purkinje cell layer demonstrated deeply stained Purkinje cells with irregular outline and invisible nucleus. Shrunken Purkinje cells also appeared. The granular cell layer revealed deeply stained cells, vacuolated areas and a vacuolated cerebellar island (Fig. 2b). Strikingly, the molecular and granular cell layers appeared normal in the tartrazine + riboflavin treated group. The Purkinje cell layer showed normal Purkinje cell with a vesicular nucleus, prominent nucleolus, and homogenous cytoplasm surrounded by Bergmann protoplasmic astrocytes. Even though, some deeply stained Purkinje cells and downward displaced Purkinje cells were seen (Fig. 2c).

### Immunohistochemical results

**Figure 3:**
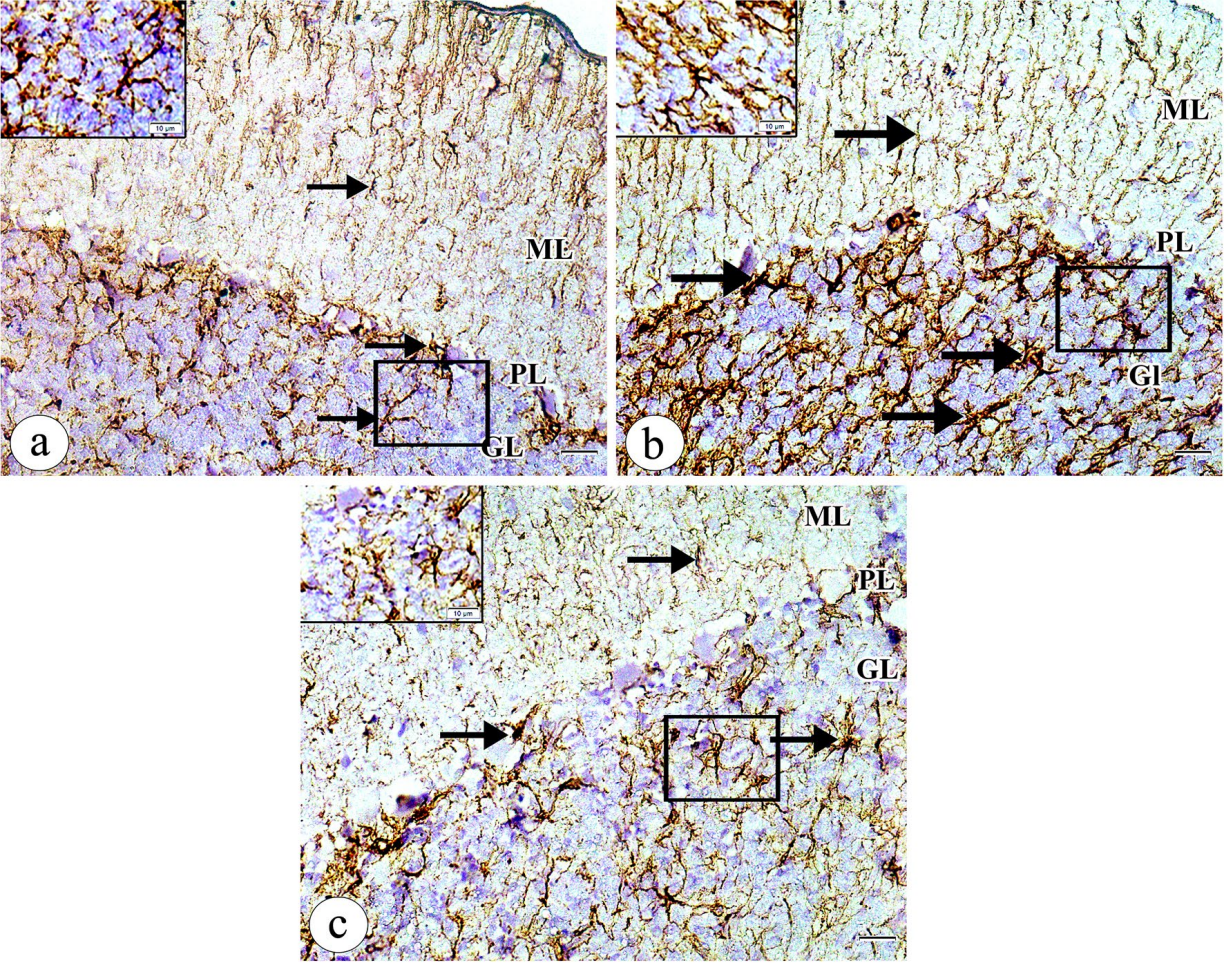
A photomicrograph of the sections of the cerebellar cortex in (**a**) the control group showing scanty mild positive GFAP immunoreactivity in the processes of the glial cells (arrow) in the molecular layer (ML), Purkinje cell layer (PL) and granular cell layer (GL). (**b**) the tartrazine treated group showing abundant strong positive GFAP immunoreactivity in the processes of the glial cells (arrow) in the molecular layer (ML), Purkinje cell layer (PL), and granular cell layer (GL). The glial cells reveal several thick branched processes when compared with controls. (**c**) the tartrazine + riboflavin treated group showing moderate positive GFAP immunoreactivity in the processes of the glial cells (arrow) in the molecular layer (ML), Purkinje cell layer (PL), and granular cell layer (GL). (GFAP counterstained with Mayer's haematoxylin, ×400, scale bar = 20 μm, inset: ×1000, scale bar = 10 μm ).

To demonstrate the astrocytes, GFAP-immunostaining of the cerebellar sections was done. In the control rats, we found scanty mild positive GFAP immunoreactivity in the processes of the glial cells in the molecular, Purkinje cell, and granular cell layers (Fig. 3a). However, the tartrazine-treated group revealed abundant strong positive GFAP immunoreactivity in the processes of the glial cells in the molecular, Purkinje cell, and granular cell layers. The glial cells revealed several thick branched processes when compared with controls (Fig. 3b). Interestingly, the tartrazine + riboflavin treated rats showed moderate positive GFAP immunoreactivity in the processes of the glial cells in the molecular, Purkinje cell, and granular cell layers (Fig. 3c).

### Electron microscopic results

**Figure 4:**
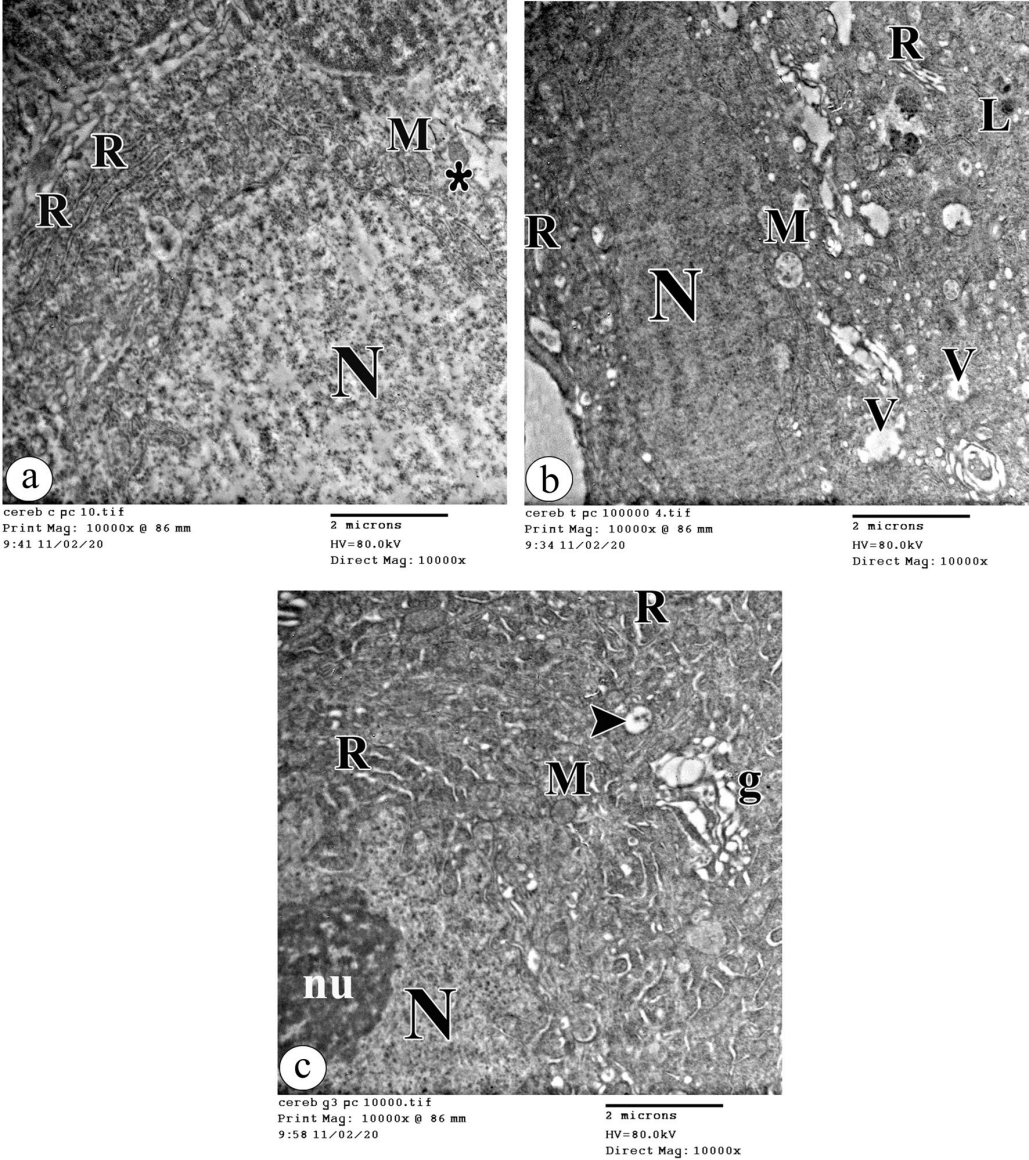
An electron photomicrograph of the Purkinje cells in (**a**) the control group showing a part of the infolded euchromatic nucleus (N). The cytoplasm contains scattered cisternae of the rough endoplasmic reticulum (R), free ribosomes (asterisk), and intact mitochondria (M). (**b**) the tartrazine treated group showing a shrunken Purkinje cell with a peripheral ill-defined electron-dense nucleus (N). Dilated cisternae of the rough endoplasmic reticulum (R), destructed electron-lucent mitochondria (M), vacuoles (V), and dark lysosomes (L) are observed in the cytoplasm. (**c**) the tartrazine + riboflavin treated group showing a part of the infolded euchromatic nucleus (N) with nucleolus (nu). Dilated fragmented cisternae of the perinuclear Golgi apparatus (g) and destructed cristae of the mitochondria (M) can be observed in the cytoplasm. Also, some cisternae of the rough endoplasmic reticulum (R) appear normal but other cisternae (arrowhead) appear dilated. (TEM, ×10000, scale bar = 2 μm).

The examination of the Purkinje cells ultra-structurally in the control group demonstrated the Purkinje cells with an infolded euchromatic nucleus. The cytoplasm contained scattered cisternae of the rough endoplasmic reticulum, free ribosomes, and intact mitochondria (Fig. 4a). On the contrary, Purkinje cells' ultrastructure was clearly altered with tartrazine treatment. The shrunken Purkinje cells with peripheral ill-defined electron-dense nuclei appeared. Also, dilated cisternae of the rough endoplasmic reticulum, destructed electron-lucent mitochondria, vacuoles, and dark lysosomes were observed in the cytoplasm (Fig. 4b). Remarkably, the Purkinje cells appeared more or less normal with infolded euchromatic nucleus and nucleolus with concomitant administration of riboflavin. Also, some cisternae of the rough endoplasmic reticulum appeared normal in the cytoplasm, but other cisternae still appeared dilated. Moreover, dilated fragmented cisternae of the perinuclear Golgi apparatus and destructed cristae of the mitochondria were observed (Fig. 4c).

**Figure 5:**
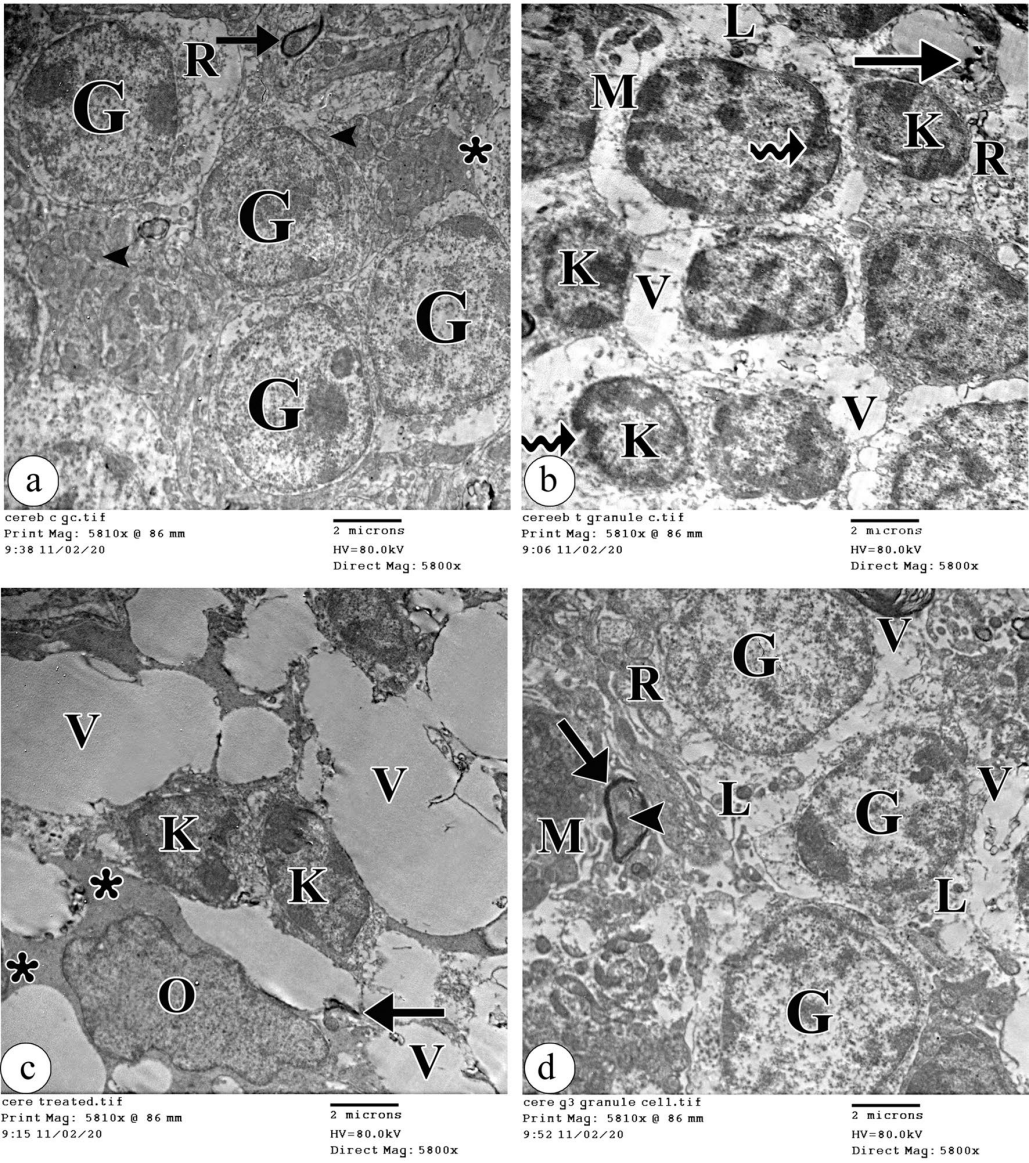
An electron photomicrograph of the granular cells layer in (**a**) the control group showing the crowded group of the granule cells (G) with central heterochromatic oval nuclei encircled by a thin cytoplasmic shell containing the rough endoplasmic reticulum (R), mitochondria (arrowhead) and free ribosomes (asterisk). The myelin sheath of myelinated nerve axons (arrow) is regularly compact. (**b**) the tartrazine treated group showing some granule cells (K) with pyknotic nuclei. Other granule cells reveal indentations (curved arrow) of the nucleolemma. The cytoplasm shows dilated rough endoplasmic reticulum (R), damaged mitochondria (M), lysosomes (L), and vacuoles (V). Disrupted myelinated fibers (arrow) are seen. (**c**) the tartrazine-treated group showing two granule cells (K) with pyknotic nuclei fused. The cytoplasm appears with many vacuoles (V) and a marked loss of cell organelles. Oligodendrocyte (O) with the process (asterisk) and degenerated myelinated nerve axon (arrow) is observed. (**d**) the tartrazine + riboflavin treated group showing restored the normal appearance of the granule cells (G). The cytoplasm shows dilated rough endoplasmic reticulum (R), intact mitochondria (M), lysosomes (L), and few vacuoles (V). The myelinated nerve axon (arrow) with intact mitochondria in the axoplasm (arrowhead) is noticed. (TEM, X5800, scale bar = 2 μm).

Regarding the granule cells, the control group showed a crowded group of the granule cells with central heterochromatic oval nuclei encircled by a thin cytoplasmic shell containing the rough endoplasmic reticulum, mitochondria, and free ribosomes in the control group. The myelin sheath of myelinated nerve axons was regularly compact (Fig. 5a). However, the tartrazine-treated group showed destructive changes in the granular cell layer. Some granule cells with pyknotic nuclei were observed. Other granule cells revealed indentations of the nucleolemma. The cytoplasm had dilated rough endoplasmic reticulum, damaged mitochondria, lysosomes, and vacuoles. Also, disrupted myelinated fibers were detected (Fig. 5b). Moreover, there was a broad spread of neuronal affection to the degree of the disappearance of some of the granular cells. Fusion of two granule cells with pyknotic nuclei, oligodendrocytes with their process and degenerated myelinated nerve axons were observed. The cytoplasm showed many vacuoles with a marked loss of cell organelles (Fig. 5c). Interestingly, the tartrazine + riboflavin treated group showed partial restoration of the normal appearance of the granule cells. The cytoplasm demonstrated dilated rough endoplasmic reticulum, intact mitochondria, lysosomes, and few vacuoles. The myelinated nerve axons with intact mitochondria in the axoplasm were noticed (Fig. 5d).

### Morphometric and statistical results

**Figure 6:**
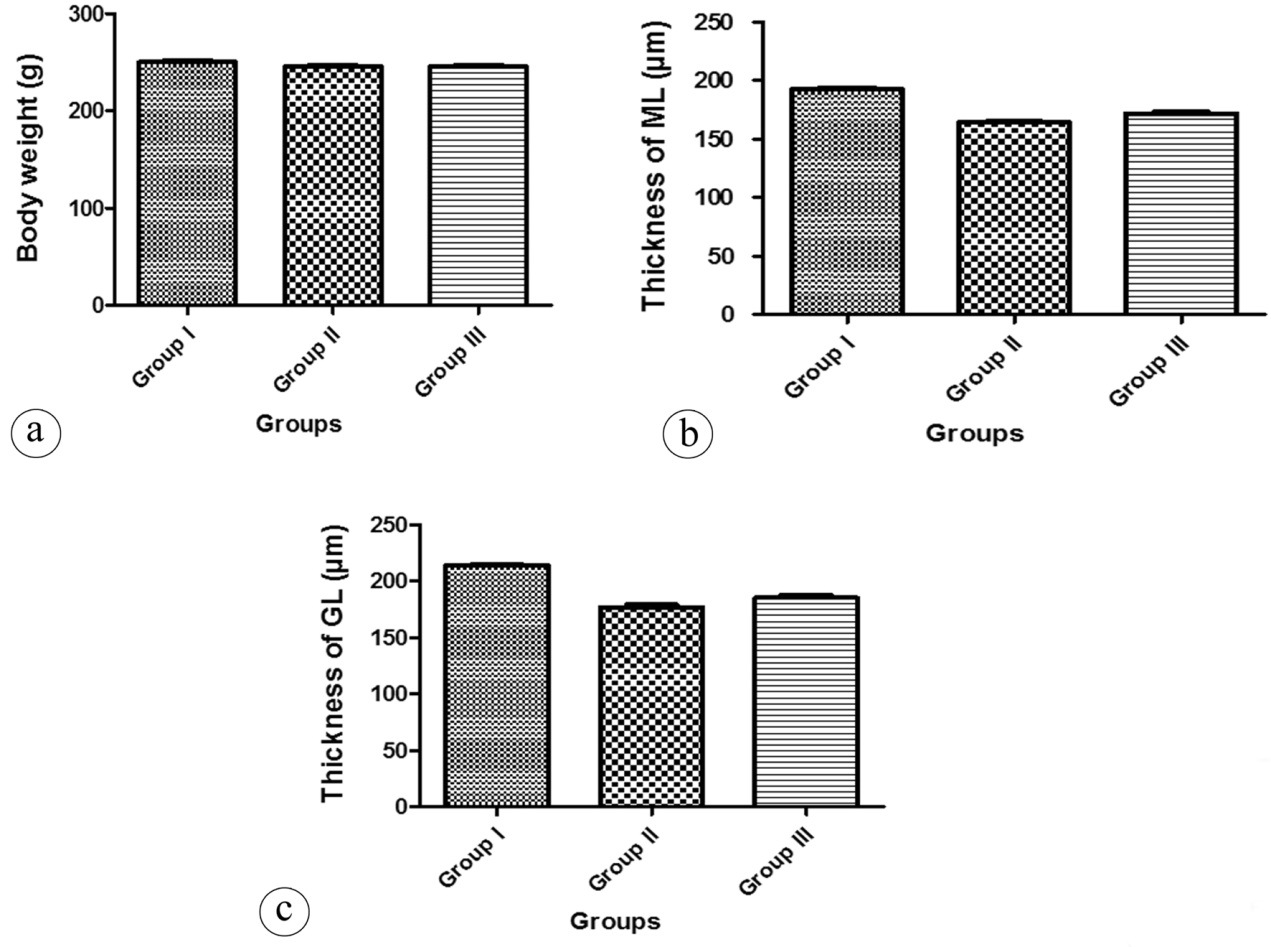
(**a**) A histogram showing the difference in the mean values of the body weight per gram in different groups. (**b**) A histogram showing the comparison of the mean values of the thickness of the molecular layer per µm in different groups. (**c**) A histogram showing the comparison of the mean values of the thickness of the granular layer per µm in different groups. The data is presented as Mean ± Standard Error.

**Figure 7:**
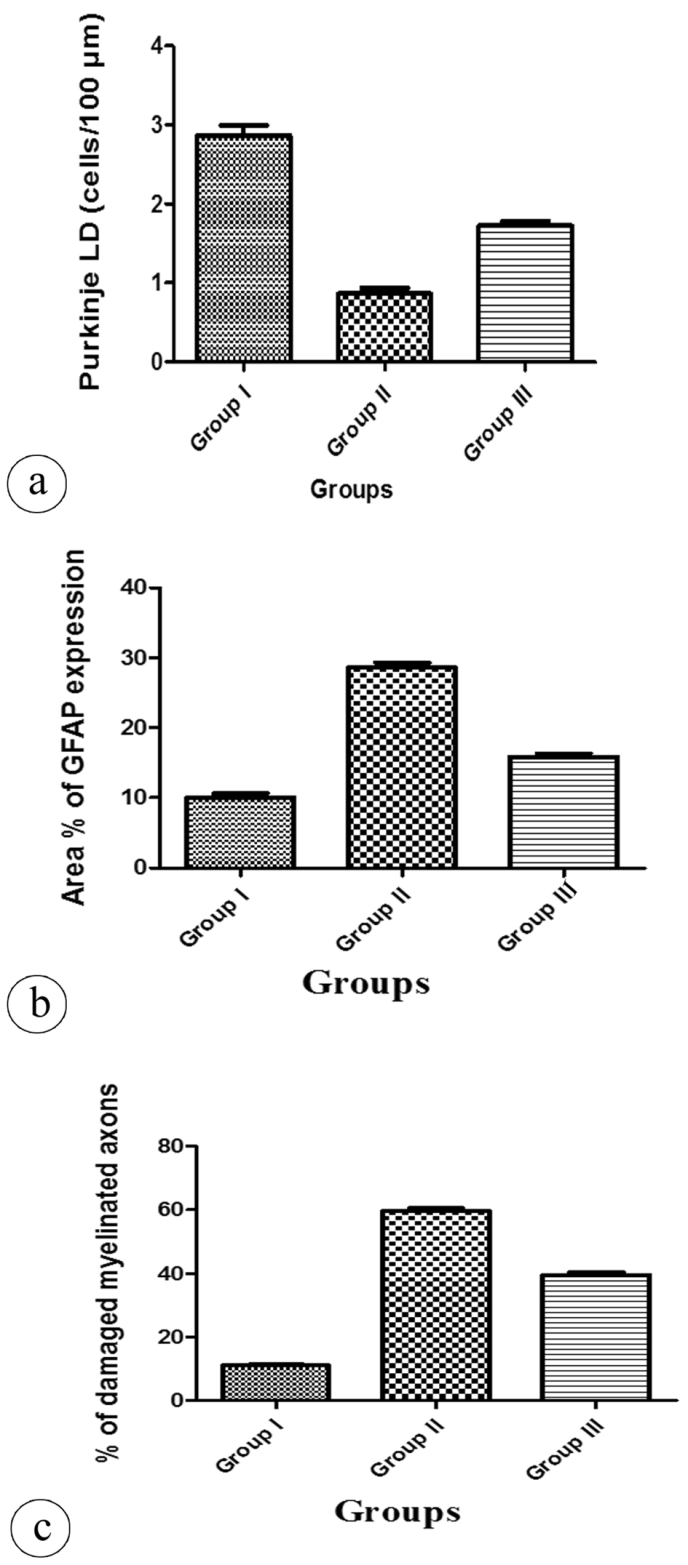
(**a**) A histogram showing the difference in the mean values of the Purkinje cell linear density (Purkinje LD) (cells/100 µm) in different groups. (**b**) A histogram showing the comparison of the mean values of the area percent (%) of GFAP expression in different groups. (**c**) A histogram showing the comparison of the mean values of the percent (%) of damaged myelinated axons in different groups. The data is presented as Mean ± Standard Error.

No statistically significant difference in the mean body weight among the experimental groups was detected (Fig. 6a, Table 1). There was a significant decrease in the mean values of the thickness of the molecular and granular layers, and Purkinje cell linear density in the tartrazine treated group when compared with the control group (Fig. 6b, c, Fig. 7a, respectively, Tables 1, 2). However, the previously mentioned parameters showed an increase in the tartrazine + riboflavin treated group which was significant as compared with the tartrazine treated group (Figs. 6b, c, 7 a, respectively, Tables 1, 2). The area percent of GFAP expression and the percent of damaged myelinated axons were increased in the tartrazine treated group in a comparison with the control group and these data were decreased in the tartrazine + riboflavin treated group. The difference between the groups was statistically significant (Fig. 7b, c, respectively, Table 2).

**Table 1:** Comparison of the body weight, the thickness of the molecular layer (ML) and the thickness of the granular layer (GL) in all studied groups.

Groups	Parameters		
	Body weight (g)	Thickness of ML (µm)	Thickness of GL (µm)
Group I	250.1 ± 5.4	192.3 ± 5.7	213.8 ± 5.3
Group II	245.5 ± 5.8	164.2 ± 3.5^a^	176.8 ± 10.8^a^
Group III	245.5 ± 4.7	171.8 ± 4.9^a,b^	185.1 ± 10.3^a,b^
*P*-value	0.06	< 0.0001*	< 0.0001*

Data are represented as Mean ± SD.

*Statistically significant difference.

^a^Statistically significant as compared with the group I,* P* < 0.05

^b^Statistically significant as compared with the group II, * P* < 0.05

**Table 2:** Comparison of the Purkinje cell linear density (Purkinje LD), the area percent (%) of GFAP expression, and the percent (%) of damaged myelinated axons in all studied groups.

Groups	Parameters		
	Purkinje LD (cells/100 µm)	Area % of GFAP expression	% of damaged myelinated axons
Group I	2.8 ± 0.2	10.06 ± 1.25	11.10 ± 0.82
Group II	0.9 ± 0.2^a^	28.60 ± 1.56^a^	59.64 ± 1.92^a^
Group III	1.7 ± 0.1^a, b^	15.83 ± 1.06 ^a, b^	39.51 ± 1.934^a, b^
*P*-value	< 0.0001*	< 0.0001*	< 0.0001*

Data are represented as Mean ± SD.

*means statistically significant difference.

^a^Statistically significant as compared with the group I, *P* < 0.05.

^b^Statistically significant as compared with the group II, *P* < 0.05.

## Discussion

Tartrazine is a widespread azoic dye included in the cosmetics, textile, pharmaceutical, and food industries. Recently, many studies have discussed the potential hazards of tartrazine for instance food allergies, carcinogenic, mutagenic, and phototoxic effects^22–24^. On the other hand, numerous studies have demonstrated that tartrazine was safe to ingest at the recommended daily consumption because neither adverse effect in humans nor in experimental models were observed^25,26^.

There is a significant role played by different cerebellar cell types in regulating motor tasks; like Purkinje cells which are responsible for information processing in motor performance and promoting motor control^27^. Therefore, investigating histopathological changes of the Purkinje cells represent a fascinating target^28^. In the light of these findings, major attention has been warranted from the researchers to seek protective agents to counteract the deleterious effects of tartrazine. However, data about the impact of tartrazine on the central nervous system structure is still far away from our knowledge. Therefore, the present study was established to assess the potential hazard of tartrazine on the cortex of the cerebellum in the adult albino rat as well as the potential safeguarding role of riboflavin.

The histological examination of the cerebellum, in the control group revealed no anomalies or alterations in histology as observed by^29^ implying that these animals were in good health and that the experiment was carried out properly.

The present study revealed marked histopathological affection of rat cerebellar cortex following tartrazine administration. These findings were consistence with the results of^30^ who declared that tartrazine intake affected the pathology of the cerebellar cortex; as it caused swelling, vacuolar degeneration, shrunken Purkinje cells, and small pyknotic nuclei. On the other hand, a previous study using different doses of tartrazine (7.5, 15,100 mg/kg) for one month concluded that the principal effects of tartrazine in the cerebellum under light microscopic examination were subcortical edema, blood vessel congestion, and cytoplasmic vacuolations of the gray matter neurons, especially in molecular and Purkinje cell layers. These pathological changes were more severe with increasing doses of tartrazine^9^. The dark cells might be one of the signs of neuronal degeneration, this is in the same line with the previous study^31^.

The semithin sections in the tartrazine treated group in this study confirmed this histopathological affection as they displayed degenerated areas in the molecular and granular cell layers. These findings came in accordance with^8,9^. Also, these findings were working side by side with the data of^32^ where the cerebral cortex of male rat pups given tartrazine orally showed many cells with pyknotic nuclei, lost cellular morphology of the cerebellum, and degeneration of the Purkinje cells.

Up to our knowledge, the current electron microscopic results are the first to report that the ultrastructural alteration of Purkinje cells and some granule cells was obvious with tartrazine treatment when compared to the control group. It was suggested that these findings in this work referred to toxicosis following tartrazine intake^33,34^. The myelin damage was detected with ultrastructural examination and classified as myelin disarrangement type III (diffused disarrangement, fusion or broken myelin sheath) as described by^35^.

These findings are in accordance with the previous studies that showed wester rats supplied with tartrazine at a dose of 500 mg/kg BW for 30 days induced splitting of the myelin sheath in the cerebrum and cerebellum^8,36^. We suggested this is attributable to direct tartrazine-induced oxidative stress or induced by nitric oxide release as previous author suggested^37^ or indirectly secondary to Purkinje cell damage.

It might be suggested that cytoplasmic vacuolations observed in this work resulted from lipid metabolism alteration, sequestration of absorbed material, endoplasmic reticulum exhaustion, autophagy, and dysfunction of the proteasome. Our explanation coincides with the studies of^38,39^. In addition, regular intracellular trafficking was dependent on cytoplasmic integrity, which was harmed by the presence of vacuolization in the cytoplasm as stated by^40^.

The mitochondrial damage and rough endoplasmic reticulum destruction observed in this work might be a reaction to chemical irritants. This hypothesis is in the same line with^41,42^ who reported that synthetic agents used for coloring food such as tartrazine, led to inhibition of mitochondrial respiration and affection of mitochondrial membranes integrity which were essential for the maintenance of vital mitochondrial functions.

Our findings support previous studies conducted by^43^ who detected that perinatal exposure to tartrazine caused histological alterations and oxidative stress in various parts of the brain and supported the role of ROS in mediating tartrazine-induced neurotoxicity.

To demonstrate the astrocytes in this study, GFAP-immunostaining of the cerebellar sections was done. The tartrazine-treated group revealed abundant strong positive GFAP immunoreactions and an increase in the area percent of GFAP expression. It was suggested that the effect of tartrazine on the cerebellum caused a boost the astrocytic activity as a protective strategy against central nervous system damage. These results coincided with^9^ who reported strong GFAP expression of the tartrazine-treated cerebellum in male albino rats as compared to the control group.

Hypertrophy and proliferation of astrocytes occurred for compensatory neuronal protection, inhibition of post-traumatic inflammatory reaction, and minimizing the damage^44^. GFAP is an intermediate filament III protein particularly located in astrocytes, enteric glial cells, and non-myelinating Schwann cells. Our immunohistochemical findings might be explained by the fact that activation of astroglial cells subsequent to the trauma of the nervous system, neurodegeneration, and exposure to any neurotoxic agent is caused mainly by activation of the GFAP gene and induction of protein. GFAP and its degradation products are rapidly liberated into biofluids after brain and spinal cord injuries, so they are considered strong biomarkers for these neurological conditions^11^. Also, GFAP increased expression had been considered one of the important neurotoxic biomarkers^45^.

In the current study, there was no change in the mean body weight among the experimental groups. These results were in accordance with former studies following tartrazine administration at different low doses of 5,7.5, 10 mg/kg/day for 90 days^46^. In the contrast, a previous study^47^ showed lower body weight gain after administration of tartrazine at a dose 15, 500 mg/kg BW orally in male rats for 30 days. Therefore, we have suggested that associated weight loss with tartrazine treatment is dose dependent. In addition, the authors^48^ suggested that the decrease in body weight might indicate toxicity and it was associated with loss of appetite and reduction in food intake.

Our morphometric studies displayed a significant diminution in the mean values of the thickness of the molecular and granular layers and Purkinje cell linear density which confirms the observed histological degenerative changes. In the light of all current results, it was suggested that tartrazine had a neurotoxic effect.

The Purkinje and granular cells vacuolation and nuclear histopathological alteration in this work indicated that tartrazine could induce oxidative stress. Therefore, the possible cause of the neurodegenerative effect of tartrazine might be contributable to generating reactive oxygen species resulting from its metabolism into one of the aromatic amines called sulfanilic acid. By interacting with nitrite or nitrate-containing meals in the stomach, sulfanilic acid can produce reactive oxygen species (ROS) as part of their metabolism. Through the metabolism of nitrosamines, reactive oxygen species such as superoxide anion, hydroxyl radical, and H2O2 may be generated, increasing oxidative stress. In pathological alterations in the brain, reactive oxygen species play a key function. This hypothesis was in coincided with^49^. Because the antioxidant defense mechanisms of the cells, such as catalase, superoxide dismutase (SOD), and glutathione (GSH) initiated its depletion as a consequence of ROS formation to hamper cell death through these toxic products, Their concentrations in the tissue homogenate dropped, especially at higher levels when there is a greater demand for them, whereas malondialdehyde (MDA) levels arise as a result of lipid peroxidation related to the effect of ROS on cellular membrane lipids^50^.

Considering riboflavin treatment, it was found that a promising protective impact of riboflavin coadministration on the cerebellar cortex in a form of dramatic improvement of degenerative and necrotic signs caused by tartrazine treatment in the present different layers of the cerebellar cortex. So, it is hypothesized that riboflavin supplementation could reduce the apoptosis and degeneration caused by tartrazine in the cerebellar cortex layers.

In the same line of the present findings, it was documented that riboflavin treatment reduced the quantity of GFAP astrocytes, reduced brain edema production, enhanced behavioral outcomes, and reduced lesion size following frontal cortical contusion injury^51^. In C57/B mice, riboflavin has been shown to protect cerebellar granule neurons against glutamate/NMDA-induced excitotoxicity in a time and dose-dependent manner^52^. Additionally, the authors^53^ demonstrated that riboflavin intake during the tartrazine recovery period resulted in an obvious significant amelioration of various light microscopic, electron microscopic histological, and immunohistochemical alterations in the gastric mucosa. Moreover, the researchers^54^ noted that there was a slowing down in hepatic tumor induction by azo-dyes in rats fed with both riboflavin and cupric oxyacetate hexahydrate. Furthermore, the authors^55^ observed the neuroprotective effects of riboflavin in mitochondrial dysfunction disorders and several neurological conditions such as neuroinflammation and glutamate excitotoxicity^56^. Moreover, riboflavin deficiency led to an increase in homocysteine or kynurenines levels which in turn caused marked neurological consequences because a vital role was played by riboflavin and the active form of pyridoxine in the metabolism of homocysteine and the pathway of tryptophan kynurenine^55^.

It has been reported that distention of rough endoplasmic reticulum cisternae is induced by accumulation of un- or malfolded protein and it is one of ER stress signs during the typical cell-death process^57,58^.We have suggested that no accumulation of malfolded protein in the normal cisterns following concomitant administration of riboflavin and it might be a sign of partial recovery. In this context, the previous researchers mentioned that there is a chance that some rER with moderate dilatation could return to normal, and the condition may be reversible^59^. These concepts need more testing because they are currently simply hypothetical.

We have suggested that the neuroprotective effect of riboflavin against tartrazine toxicity is related to its antioxidant and anti-inflammatory potency by boosting the activity of antioxidant enzymes and lowering ROS levels. Also, the protective effect of riboflavin on the cell organelles especially the mitochondria in the present study may be explained by the fact that riboflavin is a key component of the electron transport chain, the glutathione reductase, and xanthine oxidase system as well as it plays an important role in transforming glutathione from oxidized form to its reduced one^60^. Furthermore, riboflavin treatment boosted the expression of inducible nitric oxide synthase and catalase genes^61^. In both animal and human research, riboflavin shortage has been shown to impair the body's oxidative state, particularly concerning lipid peroxidation status^14^. In contrast, it was stated that the antioxidant role of riboflavin is still debated^62^. Therefore, further studies are needed to explore the effect and mechanism of riboflavin on the central nervous system.

## Conclusion

The current study displayed that tartrazine administration at an oral dose 7.5 mg/kg for thirty days to adult male albino rats induces various histopathological and immunohistochemical degenerative impacts in the cerebellar cortex. Tartrazine administration had deleterious effects on the cerebellar cytoarchitecture, and co-administration of riboflavin could alleviate the neurotoxic effects of tartrazine on the cerebellum. It was recommended usage of natural coloring food in food and medicine industries instead of tartrazine. Further studies are required to clarify the other organs affected by tartrazine as its one of the most used food ingredients.

## Data Availability

Available from the corresponding author upon reasonable request.
